# Portal-splenic-mesenteric venous thrombosis in COVID-19 patients: a systematic review

**DOI:** 10.1590/1677-5449.202301282

**Published:** 2025-02-10

**Authors:** Ricardo Zanetti Gomes, Matheus Von Jelita Salina, Vitor Hugo Moro Pironatto, Julia Kapp Lepinski, Tiago Daniel Gueiber, Beatriz Moreira Salles Juliatto, Milena Zadra Prestes, Camila Marinelli Martins

**Affiliations:** 1 Universidade Estadual de Ponta Grossa – UEPG, Ponta Grossa, PR, Brasil.

**Keywords:** SARS-CoV-2, inflammation, mesenteric ischemia, thrombosis, mesenteric venous thrombosis, splanchnic circulation, SARS-CoV-2, inflamação, isquemia mesentérica, trombose, trombose venosa mesentérica, circulação esplâncnica

## Abstract

The COVID-19 pandemic affected millions of people worldwide. In addition to respiratory impairment, this viral infection can also provoke gastrointestinal symptoms caused by vascular disorders, such as portal-splenic-mesenteric venous thrombosis (PSMVT). This systematic review aimed to investigate the profile of patients who developed PSMVT concomitant with or after viral infection and its predominant outcomes. The database searches returned 214 articles. Of these, 40 case reports were included in the review, presenting a total of 41 cases of PSMVT addressed. Males were more prevalent (n=27; 65.85%), mean age was 51.54 years, and 19.57% had a previous history of endocrine diseases. Statistically significant relationships (p<0.05) were found between patient death and tachypnea at hospital admission (p=0.043) and between patient death and age (p=0.019). It was therefore possible to identify the main profiles and risk factors for PSMVT development and mortality of COVID-19 infected patients.

## RESUMO

A pandemia de covid-19 afetou milhões de pessoas no mundo todo. A infecção pelo vírus, além de comprometer o sistema respiratório, pode promover sintomas gastrointestinais decorrentes de afecções vasculares, como a trombose venosa porto-espleno-mesentérica (TVPEM). A presente revisão sistemática teve como objetivo investigar o perfil dos pacientes que desenvolveram TVPEM concomitantemente ou subsequente à infecção por covid-19, bem como os desfechos predominantes. De 214 artigos identificados nas bases de dados, 40 relatos de casos foram incluídos na revisão, incluindo 41 casos de TVPEM. O sexo masculino foi o mais prevalente (n=27; 65,85%), com média de idade de 51,54 anos, sendo que 19,57% dos pacientes tinham doença endocrinológica prévia. Foi encontrada relação estatisticamente significativa (p<0,05) entre óbito dos pacientes e a presença de taquipneia na admissão (p=0,043), assim como entre óbito e a idade dos pacientes (p=0,019). Assim, foi possível identificar os principais perfis e fatores de risco para o desenvolvimento e mortalidade da TVPEM em pacientes com covid-19.

## INTRODUCTION

Following its outbreak in China in 2019, the pandemic caused by the SARS-CoV-2 virus has already reached over 596 million confirmed cases and continues to affect people all over the world.^1^ Although it can be an asymptomatic disease, as increasing knowledge about this disease was acquired, the most common symptomatic cases were observed to present respiratory impairment, many with pulmonary involvement. However, there are several reports of cases with involvement of other systems. In addition to the respiratory manifestations, there are also some reports of gastrointestinal symptoms, indicating damage to the patients’ intestinal mucous membrane caused by vascular disorders, including portal-splenic-mesenteric venous thrombosis (PSMVT).

Portal-splenic-mesenteric venous thrombosis is a disease characterized by obstruction of the veins of the portal system by a thrombus, such as occlusion of the portal vein, the splenic vein, the superior or inferior mesenteric veins, or their tributaries. These veins are responsible for venous drainage of the gastrointestinal tract structures such as those in the small and large intestine loops and part of the rectum. As a consequence, the clinical condition of obstruction may cause increased pressure on this venous system, with blood return to the wall and to the visceral lumen, which can result in edema, progressive impairment of the arterial circulation, ischemia, and finally, tissue necrosis in the affected segment. This can compromise the gastrointestinal tract and the individual’s general wellbeing.^2^

Taking all that into consideration, there is a general belief that the pro-thrombotic effects of COVID-19 might be directly related to its viral structure that can destabilize the renin-angiotensin-aldosterone system (RAAS) and trigger excess inflammation, platelet hyperreactivity, and endothelial malfunction, which result in coagulopathy.

The pathophysiology of COVID-19-related coagulopathy might be explained by Virchow’s triad (endothelial lesion, state of hypercoagulability, and vascular stasis).

Firstly, the endothelial lesion might be caused by SARS-CoV-2 bonding to angiotensin conversion enzyme 2 (ACE-2), expressed by endothelium, reducing angiotensin II breakdown and, consequently, favoring increased production of interleukins such as IL-6, which causes the excess inflammatory reaction with high potential to cause lesions.^2^

Secondly, a hypercoagulability state might result from the deregulated hyperinflammatory response secondary to the viral infection, and the virus spreading through the ACE-2, since high levels of Von Willebrand factor (a protein that enables platelet aggregation on blood vessel lesion sites) have been observed in severe COVID-19 cases.^2,3^

Thirdly, vascular stasis might be a consequence of the immobilization of patients severely affected by the disease. Thus, the coagulation disorder induced by the systemic inflammatory condition, endothelial malfunction, and immobilization might predispose to development of PSMVT.^3,4^

In view of the facts reported above, we understood the need to carry out this systematic review aiming to characterize those individuals who developed PSMVT simultaneous with or following the viral infection, and investigate which outcomes predominated in such cases.

## METHODOLOGY

This study is a systematic review based on the Preferred Reporting Items for Systematic Reviews and Meta-Analyses – PRISMA^5^ methodology, with registration number CRD42021286846 on the International Prospective Register of Systematic Reviews – PROSPERO protocol. Since this review used secondary data from other studies and case reports, it was exempt from the requirement for submission to or approval by the Research Ethics Committee.

The research question was: “What was the profile of patients who developed portal-splenic-mesenteric venous thrombosis simultaneous with or subsequent to COVID-19 infection and which outcomes prevailed?”. The inclusion criteria were case reports of individuals who were suspected of or affected by COVID-19 and afterwards presented coagulation disorders, thrombus, or embolus involving the portal-splenic-mesenteric system. The exclusion criteria were systematic reviews that reported only agglomerated data; reports of patients who did not present coagulation problems; and papers for which the full text was not available.

**Figure 1:**
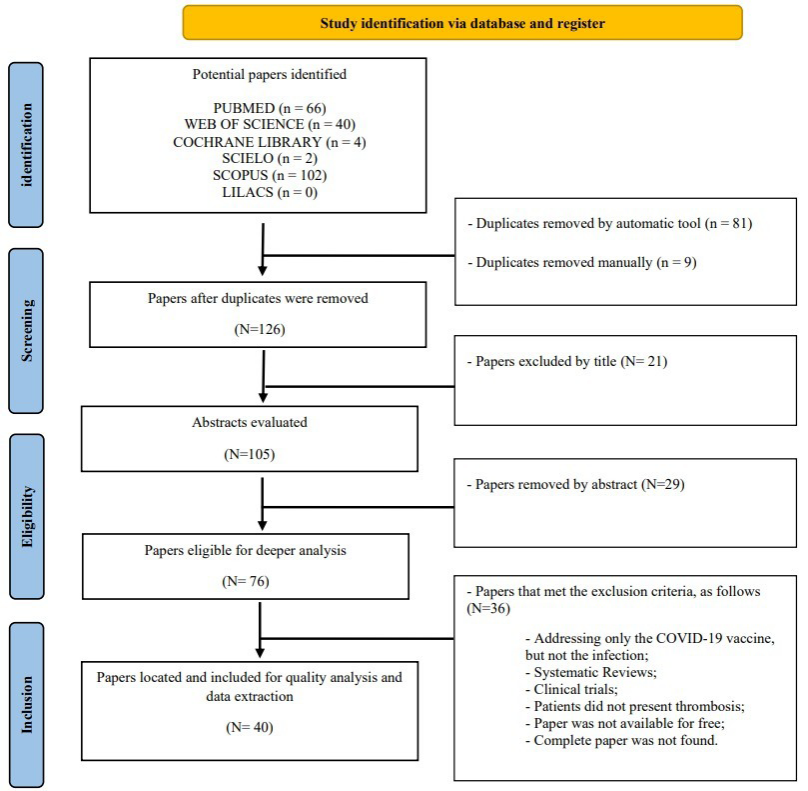
Systematic Review PRISMA Flowchart.

The search for publications was carried out in November 2021, on the PubMed (MEDLINE), Web of Science, Scopus, LILACS, Cochrane Library, and SciELO databases, with no date limits and using descriptors generated with the support of the DeCS/MesH^6^ platform and Boolean operators in English, Portuguese, and Spanish, as follows: (thrombosis OR occlusion OR obstruction OR ischemia OR hypercoagulability OR embolism) AND (venous OR vein OR vascular) AND (mesenteric) AND (COVID-19 OR SARS-CoV-2 OR coronavirus OR long-COVID OR COVID) (Figure 1).

The Mendeley - Desktop^7^ application was used to facilitate the study selection process (Figure 1), which was carried out by means of consecutive screening of the titles, abstracts, and full texts of the papers found on the databases. Finally, suitable texts were screened against the inclusion/exclusion criteria. Two independent authors conducted the study selection and data extraction collaboratively to minimize bias. Any disagreements were resolved through consultation with a third author to ensure a comprehensive and unbiased process.

Data were collected by reading the complete texts of case reports and organizing the information in spreadsheets in the Microsoft Excel program (2016).^8^ The information collected included: study year and country, patients’ age, sex, and risk factors, COVID-19 infection, analysis of signs and symptoms observed at hospital admission, COVID-19 symptoms, portal-splenic-mesenteric thrombosis/embolism observed, and description of other clinical conditions, besides prognosis or patient death.

To evaluate the risk of bias, two independent authors employed the criteria proposed by Murad et al.,^9^ which contain eight questions related to selection, verification, causality, and report, with binary answers, although not all questions are applicable to all case reports. The higher the score, the higher the methodological quality of the case report. However, we did not set a cutoff score. In instances in which consensus was not reached, a third author was consulted. Items that did not apply to the risk of bias in this review were: “Was there a challenge/rechallenge phenomenon?” and “Was there a dose-response effect?”, since they are mainly relevant in cases of medication-related adverse events. The Murad et al.^9^ method was chosen due to the absence of alternative bias assessment methods specifically tailored to case reports.

The statistical analysis was based on descriptive data analysis with simple and relative frequencies for qualitative variables and mean, median, and standard deviation and 25% and 75% percentile estimates were used for the age variable. For evaluation between outcomes, we first tested for normal distribution using the Shapiro-Wilk test, to determine use of a parametric or non-parametric approach. Next, data were analyzed using the Mann-Whitney U test. The Chi-square test or the Fisher Exact test were used evaluate differences between qualitative variable outcomes. Bar graphs were produced for better visualization of the analysis. All tests were considered significant when p<0.05 and analyses were carried out in the R 4.0.4 environment.^10^

## RESULTS

The study searches resulted in 214 papers found on the databases used. Based on the exclusion criteria, 71 papers were selected for final screening. Forty of these were case reports included in the review, describing 41 cases of portal-splenic-mesenteric venous thrombosis (PSMVT) (Figure 1).

The result of the risk of bias evaluation proposed by Murad et al.^9^ (Table 1) showed that out of the 41 case reports evaluated, 22 (53.66%) scored 6/6, 10 (24.39%) scored 5/6, and 9 (21.95%) scored 4/6, considering that 2 out of 8 criteria were not applied based on the adaptation to suit the selected case reports.

**Table 1:** Result of the risk of bias evaluation as proposed by Murad et al.^9^ (adapted as described in the text).

**PAPER**	**Criterion 1**	**Criterion 2**	**Criterion 3**	**Criterion 4**	**Criterion 7**	**Criterion 8**	**SUM**
Carmo and Cunha^11^	1	1	1	1	1	1	6
Alemán et al.^12^	1	1	0	1	0	1	4
Fan et al.^13^	1	1	1	1	1	1	6
Amaravathi et al.^14^	1	1	0	1	0	1	4
Goodfellow et al.^15^	1	1	0	1	0	1	4
Ebrahiminik et al.^16^	1	1	1	1	1	1	6
Hosoda et al.^17^	1	1	1	1	1	1	6
De Barry et al.^18^	1	1	1	1	1	1	6
Navarro-Martínez et al.^19^	1	1	1	1	1	1	6
Marsafi et al.^20^	1	1	1	1	1	1	6
Pang et al.^3^	1	1	1	1	1	1	6
Randhawa et al.^21^	1	1	1	1	1	1	6
Calcagno et al.^22^	1	1	1	1	1	1	6
Jeilani et al.^23^	1	1	0	1	0	1	4
Thuluva et al.^24^	1	1	0	1	0	1	4
Mazariegos-Rubi et al.^25^	1	1	0	1	0	1	4
Dağıstanlı and Sönmez^26^	1	1	0	1	0	1	4
Agarwal et al.^27^	1	1	0	1	0	1	4
Vidali et al.^28^	1	1	1	1	1	1	6
Mitchell et al.^29^	1	1	1	1	1	1	6
Levolger et al.^30^	0	1	1	1	1	1	5
Beccara et al.^31^	1	1	1	0	1	1	5
Cheung et al.^32^	1	1	1	1	0	1	5
Hassan et al.^33^	1	1	1	1	1	1	6
Bannazadeh et al.^34^	1	1	1	1	1	1	6
Azouz et al.^35^	0	1	1	1	1	1	5
Ucpinar and Sahin^36^	1	1	1	0	1	1	5
Dinoto et al.^37^	1	1	1	1	0	1	5
Macedo et al.^38^	1	1	1	1	1	1	6
Balani et al.^39^	1	1	1	1	1	1	6
Asghari et al.^40^	0	1	1	1	1	1	5
Abeysekera et al.^41^	1	1	1	0	1	1	5
Kukreja et al.^42^	1	1	1	1	0	1	5
Lari et al.^43^	1	1	1	1	1	1	6
Mir et al.^44^	1	1	1	0	1	1	5
Rodriguez-Nakamura et al.^45^	1	1	1	1	1	1	6
Rodriguez-Nakamura et al.^45^	1	1	1	1	1	1	6
Sehhat et al.^46^	1	1	1	1	1	1	6
Singh et al.^47^	1	1	1	1	1	1	6
Tripolino et al.^48^	1	1	1	1	1	1	6
Ignat et al.^49^	1	1	1	0	1	0	4

The following questions were answered: Criterion 1. Do the patients represent the whole investigator’s (center) experience, or the selection method is not clear to the extent that other patients with similar conditions might not have been reported? Criterion 2. Was the exposure properly verified? Criterion 3. Was the result properly verified? Criterion 4. Were other alternative causes that might explain the observation ruled out? Criterion 7. Was the follow-up long enough for the results to occur? Criterion 8. Was/were the case(s) described in enough detail to allow other researchers to replicate the study or to enable professionals to make inferences related to their own practice?

Of the whole sample selected, 28 patients presented mesenteric venous thrombosis (MVT) and 22 of these involved the superior mesenteric vein and 6 the inferior mesenteric vein, while 6 patients presented splenic venous thrombosis (SVT). We also included 14 case reports of patients who presented thrombotic obstruction of the superior mesenteric artery, 1 case of splenic artery obstruction, and 1 case of microvascular thrombosis.

### Patients’ characteristics

Male patients prevailed (n=27; 65.85%) and the mean age was 51.54 years, ranging from 28 to 84 years old. Each patient’s previous conditions were extracted, grouped, and summarized (Table 2).

**Table 2:** Patients’ previous conditions.

**Previous conditions**	**n**	**%**	**95%CI**	
Previous respiratory disorder	6	13.04	6.12	25.67
Previous psychiatric disorder	3	6.52	2.24	17.5
Previous endocrine disorder	9	19.57	10.65	33.17
Previous neurological disorder	4	8.7	3.43	20.32
Previous cardiovascular disorder	7	15.22	7.57	28.22
Previous urinary tract disorder	2	4.35	1.2	14.53

n: number of patients presenting each comorbidity; %: percentage of patients presenting each comorbidity, in relation to the total sample of 41 patients; 95%CI: upper and lower limits of each percentage for a 95% confidence interval.

In addition, other risk factors were found such as smoking habits (n=3), obesity (n=3), and pregnancy (n=1), which were not included in the grouping criteria described previously.

### Clinical conditions

Patients’ signs and symptoms observed at admission were mostly pain-related, gastrointestinal, and inflammatory. Respiratory symptoms were also reported. The patients’ clinical condition data were summarized and are presented below (Table 3).

**Table 3:** Patients’ clinical condition at admission.

**Symptom**		**n**	**%**	**95%CI**	
Pain					
Abdominal pain without irradiation		30	73.17	58.07	84.31
Abdominal pain with dorsal irradiation		3	7.32	2.52	19.43
Low-back pain irradiating to the hypogastrium		1	2.44	0.43	12.6
Abdominal pain with irradiation to the iliac fossa		1	2.44	0.43	12.6
Subcostal pain		1	2.44	0.43	12.6
Bilious colic		1	2.44	0.43	12.6
Generalized abdominal pain		1	2.44	0.43	12.6
Gastrointestinal Symptoms					
Vomiting		10	24.39	13.83	39.34
Nausea		8	19.51	10.23	34.01
Lack of appetite		6	12.2	5.32	25.54
Constipation		5	14.63	6.88	28.44
Diarrhea		4	9.76	3.86	22.55
Liquid feces		1	2.44	0.43	12.6
Ascites		2	4.88	1.35	16.14
Belching		1	2.44	0.43	12.6
Abdominal defense		1	2.44	0.43	12.6
Generalized abdominal contraction		1	2.44	0.43	12.6
Abdominal Distention		1	2.44	0.43	12.6
Rectal bleeding		1	2.44	0.43	12.6
Hematemesis		1	2.44	0.43	12.6
Absence of intestinal movement		1	2.44	0.43	12.6
Inflammatory Symptoms					
Fever		19	46.34	32.06	61.25
Prostration		10	24.39	13.83	39.34
Weakness		2	4.88	1.35	16.14
Fatigue		4	9.76	3.86	22.55
Tachypnea		5	12.2	5.32	25.54
Breaths per minute	16 to 20	3	7.32	2.52	19.43
	Over 20	3	7.32	2.52	19.43
	Up to 15	3	7.32	2.52	19.43
Tachycardia		6	14.63	6.88	28.44
Diaphoresis		3	7.32	2.52	19.43
Respiratory symptoms					
Mild bibasilar crackling		13	31.71	19.56	46.98
Crackling		1	2.44	0.43	12.6
Dyspnea		11	26.83	15.69	41.93
Dry cough		9	21.95	12	36.71
Productive cough		2	4.88	1.35	16.14
Loss of smell		2	4.88	1.35	16.14
Breathing symptoms		2	4.88	1.35	16.14
Respiratory stress syndrome		2	4.88	1.35	16.14
Others		4	9.76	3.86	22.55

n: number of patients that presented a certain sign or symptom; %: percentage of patients that presented a certain sign or symptom, in relation to the total sample of 41 patients; 95%CI: upper and lower limits of each percentage for a 95% confidence interval.

### Laboratory exams

Laboratory alterations reported included increased inflammatory markers: D-dimer (n=18), PCR (n=18), ferritin (n=8), and leukocytes (n=15). Elevation of coagulation factors was also reported: fibrinogen (n=7) and prothrombin time (n=4).|

### Imaging exams

Reports of imaging exams found included thorax CT (n=12), abdominal CT (26), abdominal angiotomography (n=6), abdominal ultrasound (n=5), and thorax teleradiology (n=3). The abdominal findings showed intestinal ischemia (n=15), intraperitoneal collection (n=5), and peritonitis (n=4), while thoracic findings revealed signs of pneumonia (n= 5).

### Treatment

**Figure 2:**
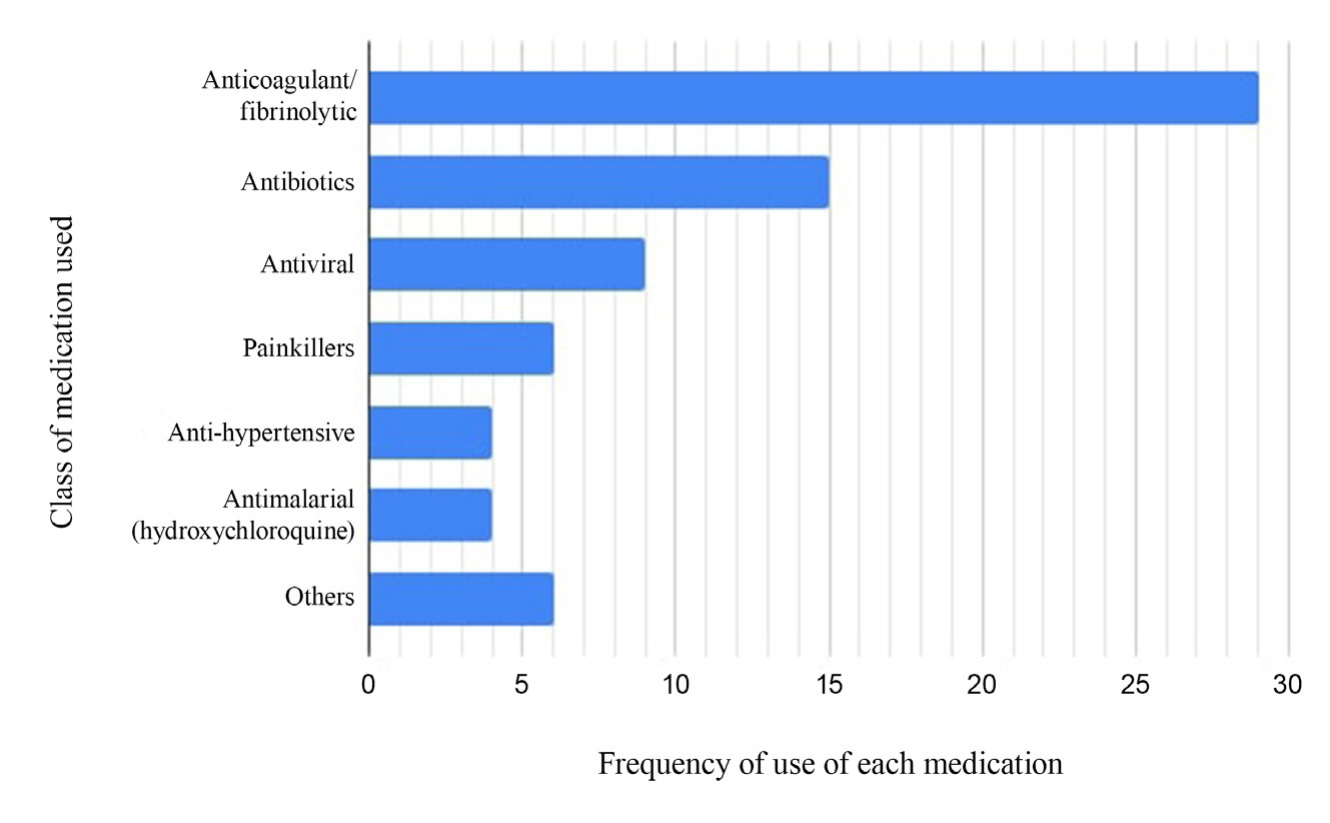
Bar chart showing the frequencies of medicines used in the patients’ treatment.

**Figure 3:**
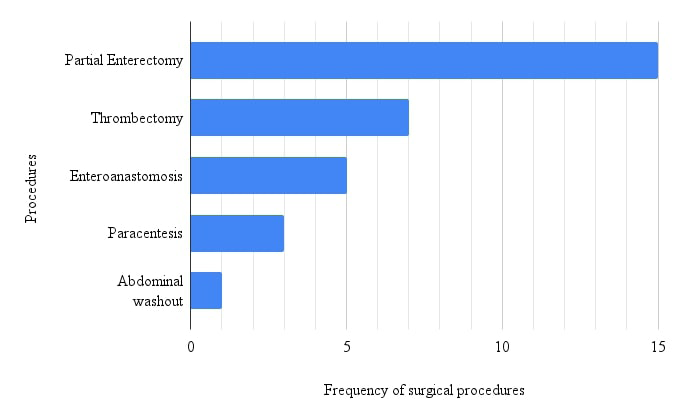
Bar chart showing the frequencies of surgical procedures described.

Patients were treated conservatively or with surgery and the results are summarized below (Figures 2 and 3).

### Outcomes

Patients’ outcome reports were summarized (Table 4). Relationships between several factors and possible outcomes such as discharge, pre-discharge death, post-discharge death, and total cure, were analyzed. Statistically significant (p<0.05) relationships were observed between patient death and tachypnea (p=0.043) at the time of admission, and between patient death and age (p=0.019). No statistically significant relationships were found between patient outcomes and any other data analyzed.

**Table 4:** Patients’ outcomes.

**Outcome**	**n**	**%**	**95%CI**	
Discharge	30	65.22	50.77	77.32
Pre-discharge death	9	19.57	10.65	33.17
Post-discharge death	3	6.52	2.24	17.5
Complete Cure	12	26.09	15.6	40.26

## DISCUSSION

This study identified characteristics that were more frequently observed in the papers analyzed and which can be considered risk factors for SARS-COV-2 patients to develop SMVT. Such features included age, usually over 50 years of age; male sex; presence of a previous endocrine disorder such as diabetes, and heart diseases, mainly hypertension and atrial fibrillation.

Other studies had already demonstrated the relationship between SMVT and previous cardiovascular disease (Strauss et al.^50^ and Guillet et al.^51^). This association is mainly due to the effects that cardiovascular diseases such as hypertension, along with the viral infection, tend to provoke on the vascular homeostatic system, promoting an inflammatory and turbulent environment that favors the emergence of a pre-thrombotic process that might lead to PSMVT.^3^

Male sex and age are also risk factors previously reported by other authors. In a review published by Dane et al.,^52^ 7 of the 9 (77.8%) patients infected by COVID-19 that ended up developing thrombosis were men. An observational study by Ahmed et al.^53^ also reported a higher prevalence of men, since 29 of the 349 (60%) cases observed were men, while only 140 were female patients. Cheruiyot et al.^54^ also identified higher prevalence of deep venous thrombosis in male and older COVID-19 infected patients. Another systematic review, by Keshavarz et al.,^55^ evaluated 22 other studies and presented similar results regarding the prevalence of male patients (n=23, 74.2%). This is probably due to the higher prevalence of cardiovascular disease in men and older individuals, who become naturally more susceptible to thromboembolic diseases such as PSMVT.

Pre-existing cardiovascular diseases were also observed in the Strauss et al.^50^ systematic review, which found evidence of a relationship between venous thromboembolism cases and these conditions. In an observational study by Omar et al.,^56^ gastrointestinal thromboembolic complications were mainly observed in patients with diabetes, dyslipidemia, hypertension, and obesity, and smokers, who were mainly men. Out of the 19 gastrointestinal thrombotic events, 17 (89.5%) were venous thrombosis. In the Pirola et al. review,^57^ 8 out of the 720 cases (1.1%) developed arterial or venous thrombosis, their mean age was 67.36 years, and six were male patients. Singh et al.^58^ presented 13 cases of acute mesenteric ischemia, 9 of whom were men, while 3 were women. The patients’ average age was 56 years and 6 patients had comorbidities such as hypertension, diabetes, and obesity. The case reported by Mir et al.^44^ described a 59-year-old, diabetic patient who developed mesenteric venous thrombosis.

Diffuse abdominal pain was the main symptom found in the cases surveyed. This occurred in a similar way to PSMVT presentation in patients who were not infected with COVID-19 and were included in a literature review by Hmoud et al.,^59^ 91%-100% of whom presented this symptom, along with sickness and vomiting, which were also found in this review.

Few of the studies selected reported on patients’ abnormal laboratory test results over the course of the disease. When reported, these changes were mainly elevated D-dimer, PCR, and leukocytes. The D-dimer change occurs due to fibrin degradation since this protein is involved in the coagulation process. Increases in PCR and leukocytes occur in the presence of any kind of inflammation. The retrospective cohort study by Guan et al.^60^ evaluated the main clinical characteristics of 1,099 patients infected with COVID-19 in China. The main laboratory findings in these patients were also D-dimer, PCR, and leukocytes.

When the diagnostic investigation imaging exams were evaluated, abdominal CT was the most used method, demonstrating intestinal ischemia in most cases and intestine wall thickness was the most common abdominal CT finding in those patients. This exam, when carried out with contrast, can identify a mesenteric venous thrombus through the presence of a hypoattenuating tubular filling defect in the affected vein in patients with acute mesenteric ischemia.^61^

The main medication therapeutic approach was the use of anticoagulant, mainly heparin (31.57%). According to the Guidelines for Diagnosis, Prevention, and Treatment of Thromboembolic complications in COVID-19 (*Diretriz sobre Diagnóstico, Prevenção e Tratamento de complicações tromboembólicas na COVID-19*),^62^ thromboprophylaxis with low molecular weight heparin (LMWH) is recommended for all patients admitted for hospital treatment when suspected of infection or infected by the virus. Other international guidelines such as those of the American Society of Hematology (2021), also recommend use of LMWH with specific criteria.^63^

Surgical procedures were used in some cases, and enterectomy was the most often used type of surgery (36.59%). Enterectomy is the most widely employed intestine resection procedure, used to manage several different situations such as intestinal obstruction, intestinal inflammatory disease, intestinal intussusception, traumatic injury, and intestinal ischemia.^64^ Since one complication of PSMVT is intestinal ischemia caused by thrombosis, mainly in mesenteric veins, enterectomy was indicated in most cases.^65^

As regards outcomes, we observed that tachypnea and age were the only factors directly related to higher mortality in PSMVT cases (p=0.043 and p=0.019). In the Bonanad et al.^66^ metanalysis, mortality was 6 times higher in COVID-19 patients over 80 years old than in younger patients. This might be due to a decrease in the body’s capability to fight the infection, along with higher prevalence of comorbidities that might worsen the condition. However, it is important to acknowledge that tachypnea and age, while significant predictors of mortality, are nonspecific clinical signs. As such, relying on these factors alone is unlikely to facilitate early diagnosis of mesenteric thrombosis, especially in a clinical context as complex as COVID-19. More specific diagnostic tools, such as angiotomography, should be employed to improve the accuracy of early detection and guide appropriate treatment strategies.

According to Harnik and Brandt,^2^ intestinal loop infarction was described as the main factor associated with death in PSMVT cases. In addition, as explained by Oldenburg et al.,^67^ the clinical presentation of patients with acute mesenteric infarction includes inflammatory signs such as tachycardia and tachypnea. This confirms the data presented in this study, in which the presence of tachypnea was related to worse outcomes for the patients. The tachypneic condition might also originate from pneumonia caused by the COVID-19 virus, which is also associated with higher mortality.^68^

As previously mentioned, there were other systematic reviews involving SARS-COV-2 infection and the development of thromboembolic events. However, the purpose of this study was to prepare a systematic review of case reports known up to this point.

Since the papers reviewed were published at the beginning of the COVID-19 pandemic, the number of studies found was limited. Moreover, during the review period, the global COVID-19 vaccination process had only recently started and the literature available at the time did not include many publications on this theme. Some studies found using the descriptors chosen for this review correlated development of PSMVT in vaccinated patients,^69-73^ but those studies were excluded from the analysis because they did not meet the inclusion criteria. Another limitation found was a lack of data on patients in the case reports, several of which were incomplete or partial, thus hampering the analysis in this review.

## CONCLUSION

Through the systematic review of case reports, we concluded that older age and tachypnea were two factors directly related to higher risk of mortality among COVID-19 infected patients who developed PSMVT. We also observed that some preexisting factors already present before the patients’ infection were characteristic of this disease, such as endocrine and cardiovascular diseases.

Systematic reviews including more recent and updated papers on this topic are needed to better identify factors related to favorable or unfavorable outcomes for patients infected with the SARS-COV-2 who developed PSMVT.
